# LIM and SH3 Domain Protein 1 (LASP-1) Overexpression Was Associated with Aggressive Phenotype and Poor Prognosis in Clear Cell Renal Cell Cancer

**DOI:** 10.1371/journal.pone.0100557

**Published:** 2014-06-23

**Authors:** Fan Yang, Xingchun Zhou, Shuangkuan Du, Yongjun Zhao, Wei Ren, Qian Deng, Fuli Wang, Jianlin Yuan

**Affiliations:** 1 Department of Urology Surgery, Xijing Hospital, Fourth Military Medical University, Xi'an, Shaanxi, China; 2 State Key Laboratory of Cancer Biology and Experimental Teaching Center of Basic Medicine, Fourth Military Medical University, Xi'an, Shaanxi, China; 3 Department of Urology Surgery, Peoples' Hospital of Shaanxi Province, Xi'an, Shaanxi, China; The Chinese University of Hong Kong, Hong Kong

## Abstract

**Background:**

LIM and SH3 protein 1 (LASP-1) is a specific focal adhesion protein that is known to be involved in numerous biological and pathological processes. LASP-1 overexpression has been described in several types of cancers, but its expression and role in clear cell renal cell cancer (ccRCC) remains unknown.

**Methods:**

Using immunohistochemistry, we analyzed LASP-1 protein expression in 216 clinicopathologically characterized ccRCC cases. We also examined LASP-1 expression in 20 paired ccRCC tissues and in 2 cell lines by real-time PCR and Western blot. Using RNA interference, we investigated the effects of LASP-1 depletion on tumor cell behavior *in vitro*. Statistical analyses were used to determine the associations between LASP-1 levels, tumor features and patient outcomes.

**Results:**

LASP-1 overexpression was observed in ccRCC tissues (*P*<0.0001) compared to adjuvant nontumorous tissues, and its expression levels were closely correlated with overall survival and recurrence-free survival (*P* = 0.044 and 0.006, respectively) in patients with ccRCC. RNA interference-mediated silencing of the LASP-1 gene in 786–0 ccRCC cells significantly inhibited cell migration.

**Conclusions:**

The results of the present study indicate that LASP-1 may serve as a prognostic biomarker for ccRCC patients and may be a promising target for the treatment of ccRCC.

## Introduction

Clear cell renal cell carcinoma (ccRCC) is a common urological malignancy worldwide[1]. Although immense improvement has been made in the treatment of ccRCC during recent years, the mortality rate of ccRCC remains high, because 40% of ccRCC patients undergoing nephrectomy will develop local recurrence or metastases[2]. Therefore, reliable prognostic biomarkers are urgently required to predict outcome and identify therapeutic targets for the treatment of ccRCC patients.

LIM and SH3 protein 1 (LASP-1) has been demonstrated to play an important role in cancer development and progression [3], [4]. LASP-1 was initially identified from a cDNA library of breast cancer metastases, and the gene was mapped to human chromosome 17q21 [5], [6]. The human LASP-1 protein contains 261 amino acids with an N-terminal LIM domain, followed by two actin-binding domains in the core of the LASP-1 protein that mediate the interaction between LASP-1 and the actin cytoskeleton at the site of cell membrane extensions, but not along actin stress fibers [7]–[10]. The SH3 domain at the C-terminus is involved in protein-protein interactions by binding to proline-rich sequences, specifically zyxin, pallidin, lipoma-preferred partner (LPP) and vasodilator-stimulated phosphoprotein (VASP) [6], [11]. LASP-1 is localized to multiple sites of dynamic actin assembly, such as focal contacts, focal adhesions, lamellipodia membrane ruffles and pseudopodia [12], [13], but the exact functions of LASP-1 are still not well understood.

LASP-1 has been reported to be overexpressed in several types of cancers and metastatic cancer cell lines, such as breast cancer [14], ovarian cancer [4] and colorectal cancer [15]. Furthermore, LASP-1 silencing in metastatic cancer cell lines resulted in a strong inhibition of cell proliferation and migration, and led to zyxin reductions at the focal contacts [4]. Interestingly, *in vitro* silencing of the LASP-1 gene reduced cell proliferation and migration and greatly affected zyxin localization [3]. In addition, gene transfection-mediated LASP-1 overexpression in SW480 CRC cells resulted in aggressive cancer cells and promoted cancer growth and metastasis [15]. However, the roles of LASP-1 in ccRCC have not been described.

In the present study, we investigated the expression of LASP-1 in ccRCC using human ccRCC tissue samples and cell lines, and assessed the association between LASP-1 expression and ccRCC outcome after resection. Moreover, we performed RNA interference (RNAi)-mediated gene silencing of LASP-1 in ccRCC cells to investigate the role of LASP-1 in ccRCC invasion *in vitro*.

## Materials and Methods

### Patients and tissue samples

Two hundred and sixteen renal tumor samples and their adjacent nontumorous tissues were obtained from patients with ccRCC who underwent radical or partial nephrectomy at the Department of Urology Surgery, Xijing Hospital, Fourth Military Medical University from August 2005 to September 2010. The diagnosis was confirmed by the postoperative pathological analysis. Patients with a history of malignancy and those who had previously received neoadjuvant therapy were excluded from the present study. No patient had detectable distant metastasis at surgery. The study population consisted of 82 women and 134 men (mean age, 63 years; age range, 18–82 years). In the present study, the clinical and pathological features were recorded. Using the 2010 TNM staging system and the Fuhrman grade classification, the tumors were classified into the following groups for the statistical analyses: early stage (TNM1 and TNM2), late stage (TNM3 and TNM4), low grade (grade 1 and 2) and high grade (grade 3 and 4). The clinicopathological characteristics of patients were retrieved from the medical records summarized in Table 1. Follow-up data were obtained by phone, letter, or the outpatient clinical database. All of the patients were followed from the date of initial surgery until either death or the closing date of this study (November 30, 2013). Recurrence was detected in 127 patients (58.7%) at the last follow-up examination, and 46 patients (31.0%) had died due to ccRCC-related disease. The mean follow-up time was 49.3 months (range, 1–67 months).

**Table 1:** Association between LASP-1 expression and clinical characteristics in ccRCC patients.

Variables	No.	LASP-1 expression		*P* value
		Low	High	
**Total**	216	124	92	
**Gender**				0.761
Female	82	46	36	
Male	134	78	56	
**Age, years**				0.606
≤63	113	63	50	
>63	103	61	42	
**Tumor size (cm)**				0.002
≤6	110	58	52	
>6	106	34	72	
**Fuhrman nuclear grade**				0.081
1/2	97	62	35	
3/4	119	62	57	
**TNM stage**				0.005
1/2	172	107	65	
3/4	44	17	27	
**Recurrence**				0.006
No	89	61	28	
Yes	127	63	64	
**Death**				0.026
No	149	93	56	
Yes	67	31	36	

Abbreviations: ccRCC, clear cell renal cell cancer; LASP-1, LIM and SH3 protein 1.

For real-time PCR and Western blot analyses, a total of 20 paired tumor tissues and matched adjacent nontumorous tissues were collected from ccRCC patients undergoing surgery treatment at Department of Urology Surgery, Xijing Hospital, Fourth Military Medical University between March and June, 2013. The 20 patients included 12 men and 8 women, with a median age of 61 years (range, 21–79 years). After resection, the fresh tissues were immediately frozen in liquid nitrogen and stored at −80°C. Both the tumor and nontumourous tissues were verified by histopathological examination.

The study was reviewed and approved by the Ethics Committee of Fourth Military Medical University, and written informed consent was obtained from all patients prior to surgery. All experimental procedures were performed in accordance with the Declaration of Helsinki.

### Immunohistochemistry

The tissue specimens were fixed in 10% formalin and routinely processed for paraffin embedding. Tissue sections (5-µm thick) were stained with hematoxylin-eosin and reviewed by two pathologists to define the cancerous and corresponding notumorous tissues. Immunohistochemistry (IHC) was performed on paraffin-embedded tumor sections using antibody against LASP-1 (1∶200, Abcam, Cambridge, UK) after antigen retrieval. A negative control without the primary antibody was prepared for all of the samples. The mean LASP-1 expression rate was assessed by inspecting at least 5 microscopic fields at 400× magnification. LASP-1 expression was considered to be negative when <10% of the cancer cells in the microscopic fields demonstrated immunostaining, and the slides were reviewed a second time to reduce the reading error.

### Cell lines

The 786–0 human ccRCC cell line was obtained from the Cell Bank of the Chinese Academy of Sciences (Shanghai, China) and cultured in RPMI 1640 medium that had been supplemented with 10% FBS. The A498 human ccRCC cell line was obtained from Tiancheng Technology Co., Ltd. (Shanghai, China) and cultured in DMEM medium supplemented with 10% FBS. The cells were harvested in the logarithmic phase of growth for use in the experiments outlined below.

### Real-time PCR

Total RNA from tumor and nontoumorous tissues of the 20 ccRCC patients was extracted using TRIzol reagent (Invitrogen, Carlsbad, California, USA) according to the manufacturer's recommendation. Reverse transcription was performed in a 20-µl reaction system with 2 µg of total RNA that had been treated with M-MLV reverse transcriptase (Promega, Madison, Wisconsin, USA) to synthesize first-strand cDNA according to the manufacturer's recommendations, followed by cDNA amplification as previously described. The primer sequences that were used for real-time PCR for LASP-1 were: (F) 5′-ATGAACCCCAACTGCGCC-3′ and (R) 5′-TCAGATGGCCTCCACGTAGTT-3′.

### Western Blot

Total protein was isolated from tumor and nontoumorous tissues of six ccRCC patients using the Total Protein Extraction Kit (KeyGen, Nanjing, China). 30 µg of protein per lane was separated using 12% sodium dodecyl sulfate-polyacrylamide gel and transferred to a polyvinylidine difluoride membrane. The membrane was blocked in 5% skim milk for 2 h and then incubated with antibody against LASP-1 (1∶1000, Abcam, Cambridge, UK) or β-actin (1∶5000, Abcam, Cambridge, UK) at 4°C overnight. After washing four times in Tris-buffered saline with Tween-20, the membrane was probed with a horseradish peroxidase (HRP)-conjugated secondary antibody (1∶2000, Proteintech Group, Chicago, Illinois, USA).

### Small interfering RNA (siRNA)-mediated LASP-1 gene silencing

Expression of human LASP-1 was knocked down using siRNA duplexes as the following sequence: 5′-AAGGTGAACTGTCTGGATAAG-3′, 5′-CUUAUCCAGACAGUUCACCdTdT-3′. Negative control siRNAs (5′-UUCUCCGAACGUGUCACGUTT-3′ and 5′-ACGUGACACGUUCGGAGAATT-3′) targeting unknown mRNA sequences were used as controls. All of the siRNAs were synthesized by GenePharma (Shanghai, China). A BLAST search of the human genome verified that the selected sequences were specific for the target genes. Cells in the exponential growth phase were plated in six-well plates at a density of 0.5×10^5^ cells/ml, cultured for 24 h and transfected with 1 µg of siRNA once they had reached 30-50% confluence according to the manufacturer's recommended protocol. Fluorescein (FAM)-labeled negative control siRNA was used to visualize the transfection efficiency.

### *In vitro* migration analysis

Cells in serum-free medium (1×10^5^ cells/200 µl) were added to the top chambers of 8-µm pore size Transwell chambers (Corning Star, Cambridge, Massachusetts, USA). The bottom chambers were prepared using 10% FBS as a chemoattractant. The cells were allowed to migrate through the porous membranes for 20 h at 37°C. The cells that had migrated through the membrane and stuck to the lower surface of the membrane were treated with a fixation/staining solution (0.1% crystal violet, 1% formalin and 20% ethanol) for visualization. For quantification, the cells were counted under a microscope in five randomly selected fields under a Nikon ECLIPS 80i microscopy at the magnification of 400×. A minimum of five chambers from three independent experiments were analyzed.

### Statistical analysis

The IBM SPSS Statistics 19.0 software was used to conduct all statistical analyses. Differences among categorical variables were analyzed for statistical significance using a chi-squared test, while quantitative variables were analyzed using the paired Wilcoxon test or unpaired *t*-test. Univariate and multivariate Cox proportional hazards analyses were used to assess the effects of various factors on prognosis. A Kaplan-Meier analysis was used to assess survival and log-rank tests were used to compare patient survival between subgroups. All *P* values were two-sided, and *P*<0.05 was considered to be statistically significant.

## Results

### LASP-1 overexpression in RCC tissues detected using IHC

**Figure 1:**
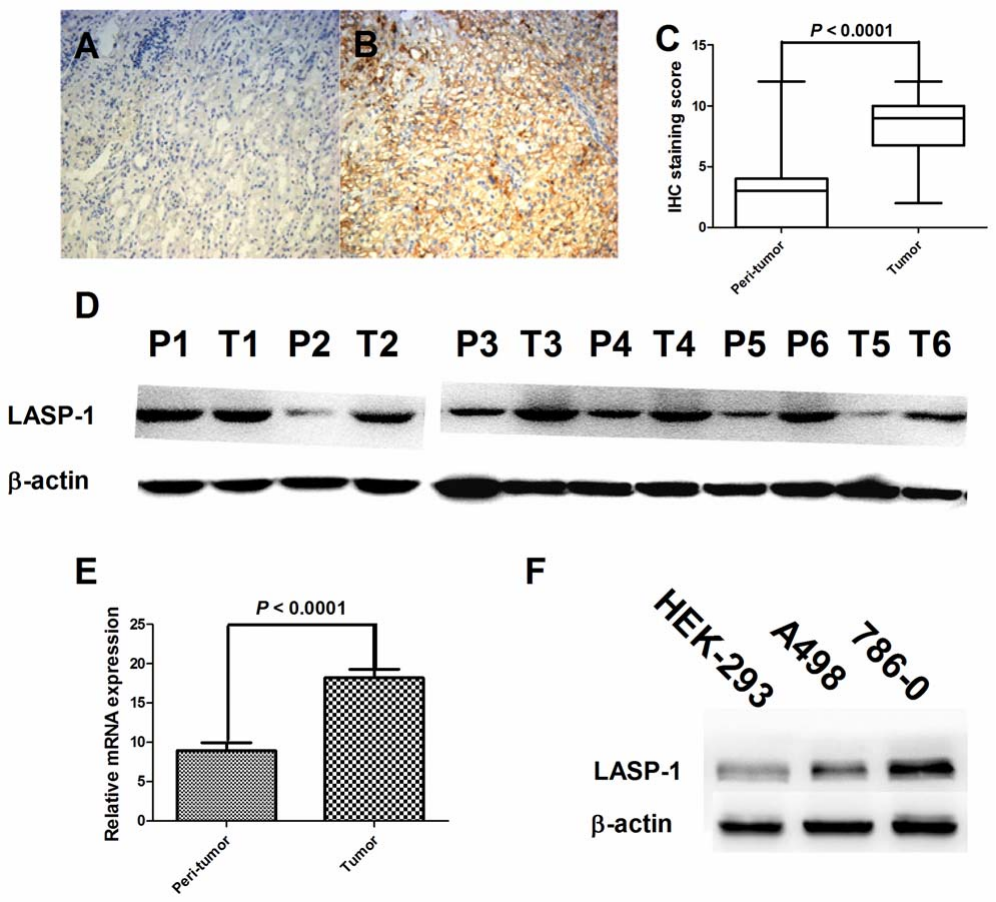
LIM and SH3 protein 1 (LASP-1) expression in clear cell renal cell cancer (ccRCC) tissues and cell lines. LASP-1 protein expression in paraffin-embedded ccRCC tissues (A) and adjacent nontumorous tissues (B) using immunohistochemistry (magnification, 100×), in which positive LASP-1 immunostaining showed brown color. Wilcoxon analysis demonstrated that tumor tissues showed significantly higher LASP-1 expression than nontumorous tissues (C, n = 216). Western blot (D) and real-time PCR (E, n = 20) analyses confirmed the findings in immunohistochemistry analysis. Western blot analysis also showed differential LASP-1 expression in human embrynal kidney cells (HEK-293) and ccRCC cell lines (F). T refers to tumor tissues, whereas P refers to peritumor (nontumorous) tissues in panel D.

To clarify the underlying role of LASP-1 in RCC progression, we first examined the protein expression level of LASP-1 using IHC in 216 tumor tissues and matched adjacent nontumorous tissues. We found that LASP-1 expression was significantly upregulated in the tumor tissues compared to the matched adjacent nontumorous tissues (*P*<0.0001; Figures 1A, B and C).

### Verification of differential LASP-1 expression using Western blot and real-time PCR analyses in tissues and cell lines

To verify the results obtained by IHC, we detected LASP-1 expression in 6 ccRCC tissues and their matched adjacent nontumorous tissues (Figure 1D). The results also revealed increased LASP-1 expression in tumor tissues compared to nontumorous tissues. Real-time PCR was then applied in 20 ccRCC tissues and paired nontumorous tissues, and LASP-1 mRNA levels were also found to be upregulated in tumor tissues (Figure 1E). Furthermore, we determined that LASP-1 was expressed in 2 ccRCC cell lines and human embryonal kidney cells (HEK-293) using western blot analysis. Consistent with the results obtained in the tissue samples, higher LASP-1 levels were detected in the more aggressive cell line (786–0) than in the low-aggressive cell line (A498) or HEK-293 cells (Figure 1F).

### Increased LASP-1 expression was correlated with tumor progression and poor prognosis in ccRCC patients

Correlations between LASP-1 expression and clinicopathological characteristics were analyzed using the chi-squared test. As summarized in Table 1, significant correlations were found between LASP-1 expression and four clinical parameters, including tumor size (*P* = 0.002), TNM stage (*P* = 0.005), recurrence status (*P* = 0.006) and death status (*P* = 0.026). The relationship between Fuhrman grade and LASP-1 expression showed borderline significance (*P* = 0.081). However, there were no statistical associations between LASP-1 expression and the remaining parameters, such as age and gender.

**Figure 2:**
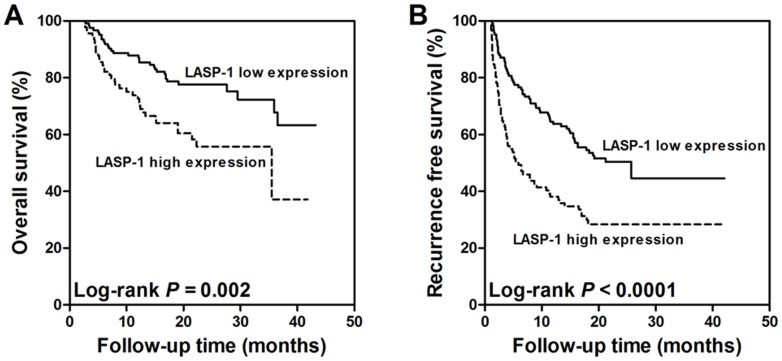
LIM and SH3 protein 1 (LASP-1) overexpression in clear cell renal cell cancer (ccRCC) is associated with poor overall survival (A) and recurrence free survival (B).

We then used univariate and multivariate Cox regression analyses to assess the association between LASP-1 expression and outcome in ccRCC patients. In the univariate analysis (Table 2), tumor size, Fuhrman grade, TNM stage and LASP-1 upregulation were significantly correlated with poor overall survival (*P* = 0.034, <0.0001, <0.0001 and  = 0.003, respectively) and recurrence-free survival (*P* = 0.005, <0.0001, <0.0001 and <0.0001, respectively) in ccRCC patients. Then, the four factors that were significantly associated with outcome (*P*<0.05) in the univariate analysis were subjected to a multivariate analysis. The multivariate analysis revealed that Fuhrman grade, TNM stage, and LASP-1 upregulation were independent prognostic factors for overall survival (*P* = 0.001, <0.0001 and 0.044, respectively) and recurrence-free survival (*P* = 0.002, 0.010 and 0.006, respectively) in ccRCC patients (Table 3). Moreover, the Kaplan–Meier curve analysis also indicated that LASP-1 upregulation was significantly associated with poorer outcome in ccRCC patients (Figure 2A and B).

**Table 2:** Univariate Cox regression analysis of the prognostic factors for ccRCC.

Variables	Overall survival		Recurrence-free survival	
	HR(95%CI)	*P* value	HR(95%CI)	*P* value
Age (>63 vs. ≤63 years)	0.91(0.56–2.49)	0.717	1.02(0.71–1.46)	0.911
Gender (male vs. female)	1.06(0.48–2.31)	0.889	1.43(0.77–2.65)	0.262
Tumor size (>6 vs. ≤6 cm)	1.71(1.04–2.79)	0.034	1.66(1.16–2.36)	0.005
Grade (3/4 vs. 1/2)	3.72(2.09–6.63)	<0.0001	2.35(1.62–3.39)	<0.0001
TNM stage (3/4 vs. 1/2)	2.05(1.60–2.64)	<0.0001	1.58(1.30–1.92)	<0.0001
LASP-1 expression (high vs. low)	2.09(1.29–3.40)	0.003	1.99(1.40–2.82)	<0.0001

Abbreviations: CI, confidence interval; ccRCC, clear cell renal cell cancer; HR, hazard ratio; LASP-1, LIM and SH3 protein 1.

**Table 3:** Multivariate Cox regression analysis of the prognostic factors for ccRCC.

Variables	Overall survival		Recurrence-free survival	
	HR(95%CI)	*P* value	HR(95%CI)	*P* value
Tumor size (>6 vs. ≤6 cm)	1.04(0.61–1.75)	0.895	1.19(0.82–1.73)	0.355
Grade (3/4 vs. 1/2)	2.77(1.49–5.14)	0.001	1.88(1.26–2.80)	0.002
TNM stage (3/4 vs. 1/2)	1.71(1.32–2.22)	<0.0001	1.32(1.07–1.62)	0.010
LASP-1 expression (high vs. low)	1.69(1.01–2.81)	0.044	1.67(1.16–2.41)	0.006

Abbreviations: CI, confidence interval; ccRCC, clear cell renal cell cancer; HR, hazard ratio; LASP-1, LIM and SH3 protein 1.

### LASP-1 silencing inhibited ccRCC cell migration *in vitro*

**Figure 3:**
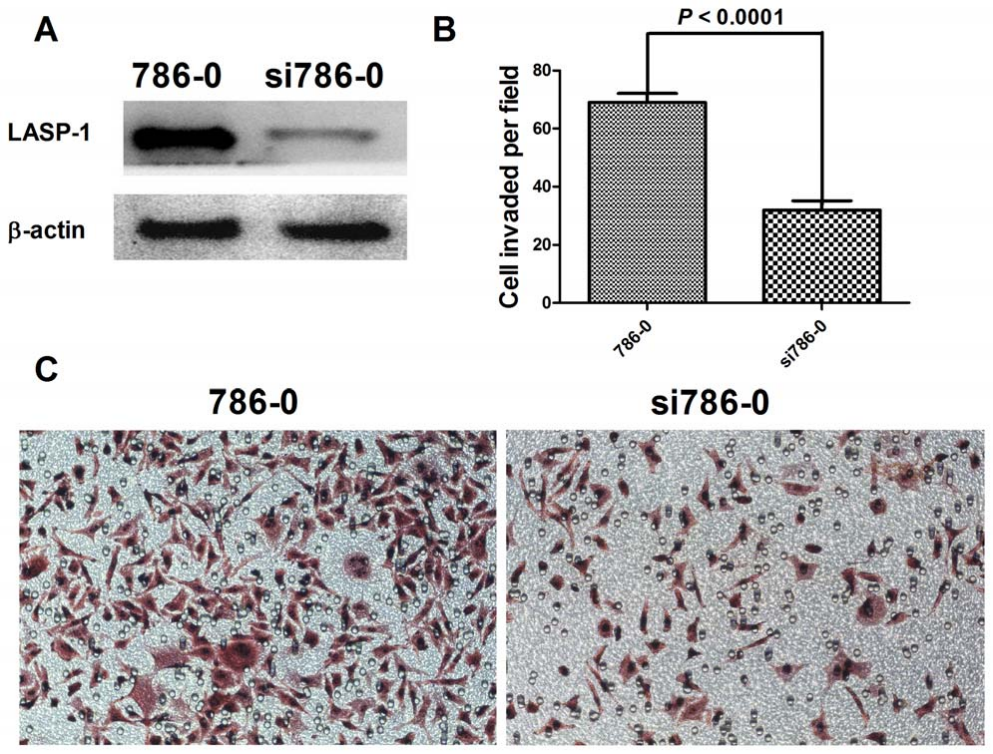
LASP-1 silencing inhibited RCC cell migration in vitro. Western blotting was used to verify knock-down of LASP-1 expression in 786–0 cells by siRNA transfection (A). An unpaired t-test was used to assess differences in the number of invaded cells per field between the 786–0 and si786–0 cell lines (B). Transwell results for the 786–0 and si786–0 cell lines are shown (C).

siRNA transfection was employed to knockdown LASP-1 expression in 786-0 cells, which displayed high endogenous LASP-1 expression. The effects of siRNA transfection on LASP-1 expression were confirmed using Western blot analysis. The amount of LASP-1 protein was obviously reduced compared to that in the negative control cells (Figure 3A). Further, transwell invasion assay revealed that silencing LASP-1 expression dramatically decreased cell mobility compared to that of control cells (Figure 3C). An unpaired *t*-test was used to assess the difference between 786–0 and si786–0 cells in the number of invaded cells per field (Fig. 3B), which revealed that the number of invaded cells per field was significantly decreased after knockdown of LASP-1 expression (*P*<0.0001).

## Discussion

In the present study, we investigated LASP-1 expression in a series of 216 ccRCC tissues and compared these data with those obtained in clinically established ccRCC for the first time. The results revealed that the protein expression levels of LASP-1 were higher in ccRCC tissues than in the paired nontumorous tissues, as indicated by IHC and validated using western blot and real-time PCR. Association analyses revealed that LASP-1 upregulation was significantly associated with larger tumor size and worse TNM stage. Taken together, these results indicated that LASP-1 may play an important role in ccRCC progression. Further prognostic analyses indicated that LASP-1 overexpression may be an independent prognosis factor in ccRCC. However, further studies with large sample sizes are required to confirm these findings and establish the role of LASP-1 in predicting the prognosis of patients with ccRCC.

Recent data have demonstrated that high LASP-1 expression in cancers is essential for cancer cell proliferation, progression and metastasis [16]. Cells with high LASP-1 expression displayed stronger vitality and were more susceptible to forming metastatic lesions due to their enhanced ability to form clones [17]. Previous studies have reported that silencing LASP-1 expression in breast, ovarian and colorectal cancer cell lines leads to reduced cell proliferation and migration [4], [14], [15]. Consistent with the results of those studies, the results of the present *in vitro* experiments in ccRCC cell lines indicate that LASP-1 expression is necessary for cell migration.

The mechanism by which LASP-1 affects cancer cell proliferation and migration remains unclear. Cell migration and the controlled assembly and disassembly of focal adhesions are highly integrated multistep processes and central features in the molecular pathology of cancers [18]. To date, more than 50 different adhesion proteins that regulate the rate and organization of actin polymerization and focal adhesion turnover in protrusions have been identified [19], [20]. LASP-1 has been shown to interact with lipoma preferred partner (LPP) and zyxin, both of which can influence actin filament dynamics [21]. Binding occurs between the C-terminal SH3 domain of LASP-1 and the N-terminal proline-rich domains of zyxin and LPP [4]. Zhao *et al*. has reported that gene transfection-mediated LASP-1 overexpression in SW480 CRC cells resulted in aggressive phenotypes in cancer cells and promoted cancer growth and metastasis [15]. This observation underscores the importance of LASP-1 in cancer.

Recent studies have shown that LASP-1 is transcriptionally upregulated in response to the morphogen Sonic Hedgehog [22]. Disruption of the Hedgehog signaling cascade leads to a number of developmental disorders and plays a key role in the formation of a range of human cancers. In this context, it is interesting to note that zyxin has also been identified as a differentially transcribed gene in several types of cancers using microarray technology [15]. Grunewald *et al*. has reported that LASP-1 overexpression mediates human ovarian cancer cell migration and proliferation and influences zyxin localization [4]. Zyxin is localized primarily at focal adhesion plaques and plays a central role in actin filament polymerization in mammalian cells. Zyxin silencing in HeLa cells results in significantly reduction in actin stress fiber formation, whereas under cyclic stretch, zyxin only dissociates from focal contacts and accumulates in the nucleus, without affecting vinculin or actin filaments [6]. The decreased cell motility after LASP-1 silencing can be explained by the functional loss of zyxin as a scaffolding protein that facilitates the formation of molecular complexes, thereby promoting site-specific actin [8]. In the present study, we knocked down LASP-1 expression in the 786-0 cell line and observed poor migration ability in vitro, which was consistent with the results of previous studies.

## Conclusions

In summary, we observed for the first time that LASP-1 was upregulated in ccRCC, implying that its important role in the development of ccRCC. LASP-1 overexpression was associated with larger tumors and aggressive phenotypes in ccRCC, and silencing of LASP-1 expression inhibited cancer cell migration *in vitro*. Therefore, LASP-1 may be a novel prognostic biomarker and a promising therapeutic target for ccRCC.
